# The relationship between food addiction and patterns of disordered eating with exercise dependence: in amateur endurance athletes

**DOI:** 10.1007/s40519-019-00794-6

**Published:** 2019-10-25

**Authors:** Carolin Hauck, Melanie Schipfer, Thomas Ellrott, Brian Cook

**Affiliations:** 1 Institute for Nutrition and Psychology at the University of Goettingen, Humboldtallee 32, 37073 Göttingen, Germany; 2 Martin-Luther-University Halle-Wittenberg, Universitaetsplatz 10, 06108 Halle (Saale), Germany; 3 Profusa Inc., 345 Allerton Avenue, South San Francisco, CA 94080 USA; 4 Alsana: An Eating Disorder Recovery Community, 2545 W Hillcrest Dr, Suite 205, Thousand Oaks, CA 91320 USA

**Keywords:** Food addiction, Yale Food Addiction Scale 2.0, Eating disorders, Eating Disorder Diagnostic Scale, Exercise dependence, Amateur endurance athletes

## Abstract

**Purpose:**

Examine the prevalence and potential relationships among food addiction (FA)—as measured by Yale Food Addiction Scale 2.0 (YFAS 2.0), eating disorders (ED)—as measured by Eating Disorder Diagnostic Scale (EDDS)—and exercise dependence (EXD)—as measured by Questionnaire to Diagnose Exercise Dependence in Endurance Sports (FESA), for the first time worldwide, in amateur endurance athletes.

**Methods:**

A total of 1022 German-speaking endurance athletes (44% male, *Ø* 36 years, *Ø* BMI 23 kg/m^2^) replied to an online questionnaire consisting of demographics, related parameters, and the German versions of YFAS 2.0, EDDS, and FESA.

**Results:**

Prevalence of FA, ED, and EXD was 6.2, 6.5, and 30.5%. The probability for FA increases with BMI, thoughts about food and EXD score, and decreases with age and when an ED is present. People with FA and people with ED vs. people with both, FA&ED, differed significantly in this cohort. Strong significant relationships were found between FA and EXD (*X*^2^ (1) = 15.117, *p* < 0.001, *n* = 1022).

**Conclusions:**

A considerable number of amateur endurance athletes may suffer from FA. The association between FA and EXD is stronger than between ED and EXD, indicating FA as a potentially more relevant subject—than ED—for prevention or therapy in people with EXD. Further studies are needed to investigate parameters and relationships between the possibly involved types of ED, FA, and EXD.

**Level of evidence:**

Level III, well-designed cohort analytic study.

## Introduction

### The concept of food addiction

The concept of ‘Food Addiction’ (FA) has gained much research and clinical attention. Specifically, similarities in food intake and consumption of drugs of abuse have led to the theory that some naturally non-occurring, highly processed foods with added fats and/or refined carbohydrates may possess an addictive potential (e.g., biscuits, chocolate, pizza; [1–3]). The substance dependence diagnostic criteria from DSM-5 [4] were applied to food, and the phenomenon of addictive-like eating was named ‘FA’ [1]. However, little consensus exists regarding etiology, clinical presentations, biomarkers and physiological explanations, and effective treatment approaches [5]. Moreover, confusion exists concerning whether FA more closely represents an emerging form of behavioral addiction [6], food-type specific form of chemical dependence [7], or an emerging subtype of a clinical eating disorder (ED; the term ED refers to a clinical eating disorder) [8]. A significant amount of substance dependence diagnostic criteria, according to DSM-5 [4], seem to be similar to those criteria meeting for ED, like binge-eating disorder (BED; [9]. Therefore, a potential overlap of 50–95% between FA and ED has been hypothesized [10–12]. However, the character of the potential overlap remains unclear [10–14]. Thus, distinguishing similarities and differences among FA from established criteria for the different types of ED is of both clinical and research importance.

### Eating disorders in athletes

The prevalence of all EDs (e.g., Anorexia Nervosa, Bulimia Nervosa) is 3–5% in Germany [15]. Athletes represent a group that is at elevated risk for specific types of ED, such as Anorexia Nervosa [16]. The prevalence of ED in elite athletes varies from 6 to 45% in female and from 0 to 19% in male elite athletes [16]. Especially in leanness- and weight-dependent sports, like endurance sports, elevated rates of ED can be found [17, 18]. For this reason, athletes represent a good group to find out potential associations between ED and FA.

### Exercise dependence in athletes

In general, physical activity is seen as a positive behavior with favorable effects on health. Nevertheless, the positive benefits of exercise can have detrimental effects when taken to excess [19] and become a compulsive behavior with harmful consequences to the individual [20]. In addition to the higher prevalence of ED in athletes, some athletes, especially endurance athletes, comprise a group which is furthermore at higher risk for harmful excessive exercise, the so-called exercise dependence (EXD) [21]. Prevalence for EXD is 0.5–3.5% in the general population [22] and up to > 50% within triathletes [23]. Some research groups also describe pathological excessive exercise as ‘exercise addiction’ [24–32]. Prevalence for exercise addiction is 0.3–42% in various samples [25, 30]. Both terms, EXD and exercise addiction may in fact represent a similar, or even the same, phenomenon of pathological excessive exercise [33, 34]. To simplify it in this paper, only the term ‘EXD’ is used, which most likely describes both EXD and exercise addiction.

Basically, athletes, especially endurance athletes, are obliged to perform excessive amounts of sport to improve their athletic performance. That may be why it is difficult to detect the line between healthy extreme exercise and pathological excessive exercise. Furthermore, especially in endurance sports, many athletes are similar to individuals with specific forms of ED, e.g., Anorexia Nervosa, in that they maintain a low body weight and low body fat mass, although they are generally otherwise healthy with healthy eating habits. Thus, a distinction between healthy exercise, as well as healthy eating habits, and unhealthy body size and athletic exercise that possibly indicated disordered eating is difficult to identify. For example, the simple carbohydrates required to fuel endurance-type athletic events may be similar to the patterns of eating defined as FA and foods and quantities typically consumed during objective binge episodes. Therefore, there is the potential for the development of FA in this unique population that is also at-risk for EXD [19] and ED [16]. To date, the relationships among these risks have not been examined. For this reason, athletes represent a good group to find out potential associations between EXD, ED, and FA.

### Known links between ED and EXD in athletes

Previous studies have already found potential links between ED and EXD [35, 36]. Research suggests similarities in etiology for excessive exercise and eating disordered attitudes and behaviors [37, 38]. The prevalence of excessive exercise was found to be nearly 40% in patients with specific forms of ED [39]. Excessive exercise was also associated with greater severity of ED symptoms [39]. Meyer and colleagues proposed that a persistent drive for physical activity represents one significant characteristic of many eating disordered patients [20]. Moreover, pathological attitudes and cognitions about exercise (e.g., EXD) may best explain the exercise and ED association [19, 28, 33, 40]. Several studies have found that EXD may be a potential mediator of the exercise and ED association in individuals with ED [33, 41, 42]. Reduced EXD symptoms have also been associated with ED symptom reduction [19, 43, 44], thus suggesting a common etiological function. Nevertheless, it is not yet examined which types of ED patients are most vulnerable for EXD. Nor was it examined whether FA, as a possible subtype of an ED [8], could be linked to EXD.

### Purpose of the study

The severe nature of the observed association between ED and EXD warrants further research on specific populations that may experience ED and EXD [20, 45]. A potential relationship between ED, EXD, and in particular FA has not yet been investigated in amateur endurance athletes. Therefore, the purpose of this study was threefold. First, we attempt to establish prevalence of FA, ED, EXD, and predictors of FA in a population that is known to be at-risk for ED. Second, as FA and ED may overlap, we attempt to discern the association among FA and ED. Third, as FA and EXD both represent addictive-like disorders and addictions highly co-occur; we hypothesized that the co-occurrence of FA and EXD will be stronger than that of EXD and ED.

The work is of clinical relevance, as the relationships between ED, EXD, and FA may offer new insights in prevention and therapy of these often co-existing phenomena. If the pathological eating should represent an FA rather than an ED, a new addiction therapy approach could be developed to achieve better therapeutic outcomes for the often severely affected individuals. The work is also important for athletes and coaches, as they need to be aware of the seriousness of harmful excessive behavior accompanied by strong physical change in team members or fellow athletes. Coaches may, thus, suggest addiction therapy for those affected. It is also important that the athlete oneself is aware of the problematic relationship between excessive exercise and pathological eating and that highly processed foods may have detrimental effects for athletes.

## Materials and methods

Our study used online data collection for a cross-sectional examination of the potential associations among FA—as measured by Yale Food Addiction Scale 2.0 (YFAS 2.0), ED—as measured by Eating Disorder Diagnostic Scale (EDDS) and EXD—as measured by Questionnaire to Diagnose Exercise Dependence in Endurance Sports (FESA) in a sample of endurance athletes.

### Participants

A total of 1204 German-speaking men and women completed on a voluntary basis an online questionnaire through a secure online data collection website (e.g., https://www.soscisurvey.de). Informed consent was given by all participants and all procedures performed were in accordance with the ethical standards of the institutional research committee (Ethics Commission of the University Medicine Göttingen) and with the 1964 Helsinki declaration and its later amendments or comparable ethical standards.

The participants were recruited via an online link which was distributed through an email listserv among sports clubs throughout Germany (especially Bavaria, Baden-Wuerttemberg, Lower Saxony, and Hesse), German Facebook groups for athletes (e.g., Runner’s World Deutschland, Challenge Roth, and Achim Achilles) and word-of-mouth recommendations, reposts, and forwarding among the participants. Participation was limited to individuals aged of at least 18 years.

Previous research has found that endurance athletes at-possible-risk for exercise dependence must engage in at least 4 h of exercise per week [46]. Therefore, all participants who self-reported less than 4 h of exercise per week and data sets with missing or implausible data (e.g., body weight of 12 kg) were excluded, resulting in a sample of 1022 German-speaking participants, further referred to as amateur athletes. No further restrictions, e.g., type of sports or race, were applied, because race/cultural origin do only play a minor role in Germany and are, therefore, little significant in Germany. Anthropometric data of the sample are listed in Table 1. More than 80% of the participants self-reported regular participations in athletic competitions, events, or contests. Overall mean body mass index (BMI) for our sample was 23 kg/m^2^.

**Table 1:** Demographics and screening measures of study population (*n* = 1022)

Variable	Mean, median, standard deviation, and minimum and maximum of study variables
Sex	43.6% male, 56.4% female
Age (years)	36.44 ($\tilde{x}$ = 35.00; SD = 18.88; 18–78)
BMI (kg/m^2^)	22.83 ($\tilde{x}$ = 22.32; SD = 3.06; 14.71–45.78)
Highest body weight (kg)	($\tilde{x}$ = 6.00; SD = 9.38; 0–64)
Absence of menstruation (in women)	11.3% (*n* = 115), 4.26 ($\tilde{x}$ = 2.00; SD = 6.78; 0–40)
Main discipline	60.8% running, 9.2% triathlon, 8.2% cycling, 7.1% fitness, 5.2% team sports, 9.5% other
Participation in competition (yes–no)	81.6% (yes), 18.4% (no)
Hours of exercise per week	7.85 ($\tilde{x}$ = 7.00; SD = 3.73; 4–28)
Years of exercise practiced	10.96 ($\tilde{x}$ = 8.00; SD = 9.44; 1–70)
Importance of exercise	7.82 ($\tilde{x}$ = 8.00; SD = 1.20; 1–10)
Number of diseases	0.29 ($\tilde{x}$ = 0.0; SD = 0.60; 0–4)
Thoughts about food (%)	22.16 ($\tilde{x}$ = 18.00; SD = 18.925; 0–90)

### Measures

The online survey contained questions on demographics, screening measures and the three following questionnaires which were used in their German versions:

#### Demographics and screening measures

Demographic data were collected following the three questionnaires. These were: sex, age, body weight, body size, highest body weight, absence of menstruation (in women), main discipline, competition, hours of exercise per week, years of exercise practiced, importance of exercise, and thoughts about food and number of diseases. The BMI was afterwards calculated by the authors.

#### Yale Food Addiction Scale 2.0 (YFAS 2.0)

The Yale Food Addiction Scale 2.0 [47] is a 35-item self-report questionnaire, which applies the Diagnostic and Statistical Manual of Mental Disorders (5th version, DSM-5 [4] criteria for substance-related and addictive disorders (SRAD; e.g., tolerance, withdrawal, etc.) to the consumption of foods. It is the only validated instrument to operationalize addictive-like eating behavior in humans. The threshold for an YFAS 2.0 FA is met by endorsing two or more of the 11 DSM-5 SRAD criteria, plus showing clinically significant distress or impairment. The outcome measure for the YFAS 2.0 is the number of symptoms endorsed (0–11). Within the validation study by Gearhardt et al., the YFAS 2.0 has demonstrated internal reliability (*α* = 0.90) and convergent validity with other measures of problematic eating [47]. The YFAS has been translated to German [48].

#### Questionnaire to diagnose exercise dependence in endurance sports (Fragebogen zur Erfassung des Sportverhaltens bei Ausdauersportlern; FESA)

The questionnaire to Diagnose Exercise Dependence in Endurance Sports [46] measures EXD via 16 items on a seven-point Likert scale. It includes the following five factors: expected positive consequences, interference with social life, health, withdrawal symptoms, and exercise as a possibility to compensate for psychological problems. The FESA is a new tool to screen and categorize athletes into three groups: committed to exercise, focused on exercise, and at—possible—risk of EXD. The validation study in 314 German endurance athletes showed satisfying internal consistency (*α* = 0.643–0.808) and model fit (Standardized Root-Mean-Square Residual: 0.045; Root-Mean-Square Error of Approximation: 0.035; Comparative *Fit* Index: 0.975; Tucker Lewis Index: 0.986) [46].

#### Eating Disorder Diagnostic Scale (EDDS)

The Eating Disorder Diagnostic Scale is a 22-item brief self-report questionnaire. It may separate between potential risks for anorexia nervosa, bulimia nervosa, binge-eating disorder, or eating disorders not specified [49]. Response formats vary from Likert to yes–no, frequency and write-in formats, to assess all DSM-IV diagnostic symptoms for the three ED [50]. The scale is reliable (*r* = 0.87) and internal consistent (mean *α* = 0.89). According to the development of the fifth version of DSM [4], the EDDS was adapted and resulted in a 23-item scale, which was translated in German language [51]. The outcome measure for the EDDS is the presence or absence of an ED (yes–no).

### Procedure

All study measures were completed through a secure online data collection website (e.g., https://www.soscisurvey.de). The webpage reached 10,756 clicks within the month of the survey. A total of 1204 people completed the full questionnaire in this period of time, resulting in a sample of 1022 participants who exercise at least 4 h per week, and were referred to as amateur athletes.

### Statistical analyses

Data were analyzed using SPSS 24 and 25. Descriptive analyses include mean, median, standard deviation, and minimum and maximum of study variables. Prevalence was assessed using syntax of YFAS 2.0, EDDS, and FESA. To test the relationship between the binary dependent variable FA (yes/no) and independent demographic, eating, and ED and EXD-related variables (e.g., predictors), a binary logistic regression analysis was conducted. To test potential overlaps or differences between FA (binary) and ED (binary), a one-way ANOVA with Bonferroni post hoc tests was conducted for the groups FA, ED, and FA&ED. A power calculation showed a power of 0.998. Relationships between FA (binary) and EXD (binary) and between EXD (binary) and ED (binary) were assessed via Chi-squared tests and Fisher’s exact test.

This analytic approach allowed us to evaluate:

1. the prevalence and frequencies of possible risks for FA, ED, and EXD;
2. potential predictors of FA;
3. potential differences between people with FA and people with ED; and
4. possible associations between FA and EXD and between ED and EXD in German-speaking amateur endurance athletes.

## Results

### Prevalence and frequencies of potential risks for YFAS 2.0 food addiction, EDDS eating disorders, and FESA exercise dependence

**Fig. 1:**
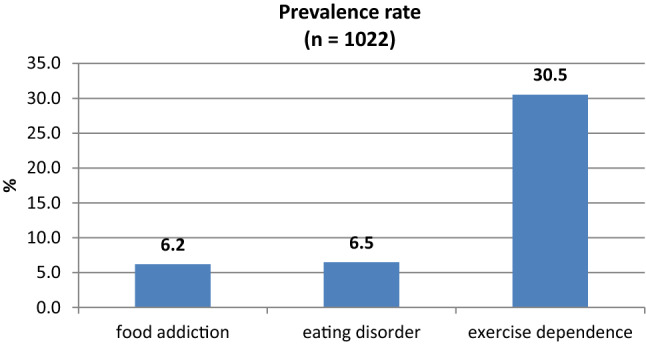
Prevalence rate of food addiction, eating disorders, and exercise dependence

Table 2 shows prevalence, frequencies of potential risks for YFAS 2.0 food addiction, EDDS eating disorders, and FESA exercise dependence and additional variables. Figure 1 shows the frequency of occurrence of FA, ED, and EXD in the whole sample.

**Table 2:** Prevalence of potential risks for FA, ED, and EXD and additional variables (*n* = 1022)

Variable	Prevalence (*n*)	Additional variables
FA	6.2% (*n* = 63)	Mean symptom count = 0.92 (SD = 1.98, range = 0–11)
ED	6.5% (*n* = 66)	Variants of ED: 0.7% anorexia nervosa 2.5% atypical anorexia nervosa 2.3% bulimia nervosa 0.3% binge-eating disorder 0.7% night-eating syndrome
	Men: 2.7% Women: 9.4%	
EXD	30.5% (*n* = 312)	Mean score = 24.124 (SD 4.09, range 12–38)

### Potential predictors of FA—logistic regression analysis

A logistic regression analysis revealed that the model (*X*^2^ = 122.413, *df* (12), *p* < 0.001, *n* = 1022) was significant. The explained variance was 45.2% (R-square according to Nagelkerke = 0.452). The Wald test revealed that the predictors (age, BMI, thoughts about food, and presence of an ED and EXD total score) were significant, with no significant influence of sex, number of diseases, absence of period, years of sports, participation in competitions, importance of sports, and hours of sports per week. The probability of a risk for FA increases by 14.1% with each unit of BMI, by 2.5% with each percentage of thoughts about food, by 15.3% with each unit of EXD score, and decreases by 5.7% with age and by 93.2% with presence of an ED:

$$\begin{aligned} {\text{Logit}} = & - 6. 1 7 3\, + \,0. 9 1 1*{\text{sex}}{-}0.0 5 8*{\text{age}}\, + \,0. 1 3 2*{\text{BMI}}\, + \,0. 4 1 2*{\text{amount of diseases}} \\ & + \,0. 80 3*{\text{absence of menstruation}}\, + \,0.0 2 5*{\text{thoughts about food}} \\ & + \,0.0 2 8*{\text{years of sports}}\, + \,0. 9 30*{\text{participation in competitions}}{-}0.0 5 1 {\text{ importance of sports}} \\ & {-}0. 100*{\text{hoursof sports}} - 2. 6 8 2*{\text{ED}}\_{\text{binary}}\, + \,0. 1 4 2*{\text{EXD score}} .\\ \end{aligned}$$

### Differences between people with FA and people with ED

One-way ANOVA with Bonferroni post hoc tests were conducted to determine differences between people with FA (*n* = 31), people with ED (*n* = 34), and people with FA&ED (*n* = 32). The three sub-groups differed significantly in the variables sex and number of symptoms, with no difference in age, BMI, absence of menstruation, thoughts about food, years of sports, participation in competition, importance of sports, and hours of sports (Table 3).

**Table 3:** Significant differences between the three sub-groups people with FA, people with ED, and people with FA&ED (*n* = 97)

Variables	Significant difference	People with FA (*n* = 31)	People with ED (*n* = 34)	People with FA and ED (*n* = 32)
Sex	(*p* < 0.05)	9 male, 22 female	10 male, 24 female	2 male, 30 female
Age	(n.s.)			
BMI	(n.s.)			
Absence of menstruation	(n.s.)			
Number of diseases	(*p* < 0.05)	18 none, 13 ≥ one	23 none, 11 ≥ one	14 none, 18 ≥ one
Thoughts about food	(n.s.)			
Years of sports	(n.s.)			
Participation in competition	(n.s.)			
Importance of sports	(n.s.)			
Hours of sports	(n.s.)			

One-way ANOVA showed that all three sub-groups differed significantly in the following variables: amount of thoughts about food (% per day) (*F*(2,93) = 4.284, *p* < 0.05, *n* = 95) and number of diseases (*F*(2,94) = 3.906, *p* < 0.05, *n* = 96). Bonferroni post hoc tests showed that the differences occurred between the following groups:

- FA vs. FA&ED: thoughts about food M(FA) = 36.03), *M*(FA&ED) = 51.94), *p* < 0.05); number of diseases M(FA) = 0.42, M(FA&ED) = 0.97, *p* < 0.05),
- ED vs. FA&ED: thoughts about food M(ED) = 37.71), *M*(FA&ED) = 51.94), *p* = 0.05).

There was no significant difference for age, BMI, years of sport, and importance of sport and hours of sport per week between the groups.

### Associations between FA and EXD, and between ED and EXD

A significant relationship between FA and EXD (*X*^2^(1) = 15.117, *p* < 0.001, *n* = 1022, Fisher’s exact test: *p* < 0.001) was observed (CC = 0.121, *p* < 0.001, Cramers *V* = 0.122, *p* < 0.001).

A significant relationship between EXD and ED (*X*^2^(1) = 10.727, *p* < 0.01, *n* = 1022 Fisher’s exact test: *p* < 0.01) was observed; however, this relationship was not very strong (CC = 0.102, *p* < 0.01, Cramers *V* = 0.102, *p* < 0.01).

## Discussion

### Prevalence and frequencies of potential risks for YFAS 2.0 food addiction, EDDS eating disorders, and FESA exercise dependence

#### YFAS 2.0 food addiction

Prevalence of FA was comparable to a representative study within the German population (7.9%; [52] and within the range of previous population studies (5–10%; [53–55]), indicating that amateur endurance athletes represent a group which is at comparable possible risk for FA as the general population. This finding is important, because most endurance athletes naturally need large quantities of high-energy food, often with high amounts of added fat/sugar, to fuel their high-energy requirements and/or maintain or improve athletic performances. In non-athletes, these high-fat, high-sugar foods appear to be particularly associated with FA [3]. Most athletes (> 90%), nevertheless, do not suffer from FA. However, it seems that there are a small proportion of athletes who suffer from a loss of control over and effort to an unsuccessful cut-down on eating. These athletes may develop problems managing a balanced caloric intake and thereby be at increased possible risk for FA. Our results suggest that there are a sizable proportion of vulnerable athletes who shows pathological eating behavior and may be in need of intervention. Conversely, it is possible that the YFAS 2.0 may not be feasible for normal weight athletes, as the YFAS was first developed to explain the rising prevalence rates of obesity in obese individuals [1]. Further research is needed to examine the impact of high amounts of high density foods, i.e., foods containing large amounts of simple/complex carbohydrate and fat in this unique population.

#### EDDS eating disorders

The prevalence of ED—as measured by EDDS—was with 6.2%, as expected, higher in this sample of athletes than in the European general population (~ 1%) [56]. Prevalence of ED in athletes ranks between 0 and 45% [16, 17]. The current study results fit within this range. According to Sundgot-Borgen and Torstveit, athletes diet and use extreme weight control methods to increase performance [17] as extra body weight can limit performance [57]. This can result in pathological eating. Our study confirms that still today, German endurance athletes represent a population that is at increased risk for ED.

#### FESA exercise dependence

The prevalence of EXD—as measured by FESA—was 30.5% which is higher in this athlete sample than in the general population (0.5–3.5%) [22]. This was an expected result as most of the current participants were performance-oriented endurance athletes (triathletes and runners) and these athletes are among the risk-takers for EXD [58, 59]. Thus, the prevalence of EXD was comparable to earlier results in studies of athletes, which rank up to 50% [23]. Our study, therefore, confirms that endurance athletes represent a population that is at increased potential risk for EXD and warrants further research attention with a special focus on prevention and therapy.

### Predictors of FA—logistic regression analysis

Our study replicates findings of a higher vulnerability of FA in people with lower age [52, 55] and higher BMI [52, 54, 60]. The study, however, failed to report significant results for sex. This may be due to the relatively small number of people with FA with 63 affected compared to the total sample of 1022 participants. The results indicate a higher level of eating-related psychopathology in people with FA compared to people without FA [61], with a higher percentage of daily thoughts about food. Furthermore, a higher score of EXD increases the possible risk for FA, which may point to an association between FA and EXD; but interestingly not with FA and ED, as the presence of ED reduces the possible risk for a FA. Our results show that FA is more common in people with EXD than in people with ED. This may be due to the fact that people who are suffering from one addictive disorder (EXD) have an increased potential risk for addictive comorbidities (FA; [62]). Endurance exercise and eating are closely connected, since on one hand a sufficient energy supply and on the other hand a low body weight are necessary for the best performance. This can result in a vicious circle of high amounts of exercise and pathological eating. The reduced potential risk for FA when an ED is present may point to more distinct than shared parameters between the two constructs FA and ED. No significant influence of the number of comorbidities and the absence of period (in women) on FA was found. FA is not characterized by a high weight loss and a low body weight, which is often followed by absence of period/amenorrhea in women and the so-called athletes-triad and further comorbidities. In FA, however, absence of period and comorbidities does not seem to play an important role, which may further distinguish FA from ED, because amenorrhea and comorbidities do play a role in specific forms of ED, e.g., Anorexia Nervosa [63–66].

Furthermore, the presence or absence of FA was not related to the years of practiced sports, the participation in competitions, the importance of sports, and the hours of sports per week, indicating that exercise-related variables may not explain FA in endurance amateur athletes and are potentially negligible when it comes to FA in this group of athletes.

### Differences between people with FA and people with ED

The one-way ANOVA with post hoc test indicate that FA and ED may represent different phenotypes of disordered eating with differences in eating pathology, e.g., thoughts about food and number of comorbidities. This result points out that FA may differ from ED, in the population of athletes. Consequently, addictive-like eating behavior may represent a new option in the prevention and therapy of disordered eating in athletes. However, demographics, e.g., sex, age, and BMI and exercise-related parameters, e.g., hours of exercise per week, do not differ significantly between FA and ED. Even the binary regression ("Predictors of FA—logistic regression analysis" section) on FA showed no differences for sex and exercise-related parameters. This may indicate that FA and ED may share demographic parameters, and in endurance athletes also similar patterns of exercising, but distinct variables of eating pathology. Shared demographics are also found in specific types of ED, as young females in endurance sport are at elevated risk for EDs [17, 57, 67–69].

### Associations between FA and EXD and between EXD and ED

The probability of FA increased with an underlying EXD. The relationship is stronger for FA and EXD (*X*^2^ (1) = 15.117, *p* < 0.001, *n* = 1022; CC = 0.121, *p* < 0.001, Cramers *V* = 0.122, *p* < 0.001) than for ED and EXD (*X*^2^ (1) = 10.727, *p* < 0.01, *n* = 1022; CC = 0.102, *p* < 0.01, Cramers *V* = 0.102, *p* < 0.01). The relationship between EXD and ED has often been confirmed in previous studies [33, 39, 41]. Nevertheless, our study shows that the relationship between ED and EXD may be more complex than previously reported in the literature. The disordered eating behavior in athletes can potentially be better explained by addictive-like eating behavior than by classical eating disorders. This assumption could be supported by the fact that addictions often co-occur [62].

## Limitations

The study was carried out online; therefore, the information provided is all self-declaration. Self-report questionnaires and self-report anthropometric data cannot be used to draw up diagnoses (e.g., for eating disorders), and these measures permit at best the examination of ‘a possible or potential risk of FA or ED’, which in no way means the ‘diagnosis of FA or ED’. A valid diagnosis of FA or ED requires clinical evaluation as well as administration of questionnaires [4]. Future clinical studies are needed to assess ED and to further define the concept of FA [70]. Furthermore, self-report data are not indicative of the true parameters of a person (e.g., body composition). Furthermore, they are limited in their significance, especially in persons with eating disorders. Nonetheless, the BMI, which has been calculated using the data on body weight and body size provided, is considered as a valid and appropriate assessment method in online surveys [71]. An online survey was selected for a variety of reasons, e.g., the availability of a sufficient number of performance-oriented endurance athletes throughout Germany. Validated instruments were used to ensure comparability with previous studies.

### Critique of measures

However, clinical assessments are needed to more accurately assess all variables included in this study and to clinically diagnose eating disorders which in turn can contribute to a higher significance of the detected relationships between disordered eating and excessive exercising.

## Conclusion

Athletes are at increased risk of ED; this study, however, is one of the first to investigate the presence of FA in an athlete sample. This study demonstrates that some amateur endurance athletes may suffer from FA (6.2%) and that FA is not only prevalent in overweight or obese people, but also in amateur endurance athletes. The disordered eating behavior in athletes can potentially be better explained by addictive-like eating behavior than by classical eating disorders. Furthermore, the results highlight the possible relationships between FA and EXD, which is stronger than between ED and EXD, indicating a relevant subject for prevention and therapy options in athletes and points to a co-occurrence of the potential addictions EXD and FA. Physical exercise or sports is often used in the therapy of ED. Since the potential risk of EXD is increased in patients with ED or FA, prudence is advised in these persons. Further studies are needed to investigate parameters and relationships between ED, FA, and EXD.
